# Expanding malaria diagnosis and treatment in Lao PDR: lessons learned from a public–private mix initiative

**DOI:** 10.1186/s12936-017-2104-5

**Published:** 2017-11-13

**Authors:** Nouannipha Simmalavong, Sengkham Phommixay, Phoudaliphone Kongmanivong, Odai Sichanthongthip, Bouasy Hongvangthong, Deyer Gopinath, David M. Sintasath

**Affiliations:** 1 Center for Malariology, Parasitology, and Entomology, Ministry of Health, Vientiane, Lao PDR; 2 World Health Organization, Bangkok, Thailand; 3 U.S. President’s Malaria Initiative, United States Agency for International Development, Regional Development Mission for Asia, Bangkok, Thailand

**Keywords:** Public–private mix, Lao PDR, Malaria diagnosis, Treatment

## Abstract

**Background:**

As in other countries of the Greater Mekong Sub-region (GMS), the private health sector constitutes a significant avenue where malaria services are provided and presents a unique opportunity for public–private collaboration. In September 2008, a public–private mix (PPM) strategy was launched initially in four northern and southern provinces in Lao PDR to increase access to rapid diagnostic tests (RDTs) and artemisinin-based combination therapy (ACT), improve quality of care, and collect routine malaria data from the private sector. Throughout the process, key stakeholders were involved in the planning, monitoring and supervision of project sites. Following an initial assessment in 2009, the PPM initiative expanded to an additional 14 district sites to a total of 245 private pharmacies and 16 clinics covering 8 provinces and 22 districts. By June 2016, a total of 317 pharmacies, 30 clinics in 32 districts of the 8 provinces were participating in the PPM network and reported monthly malaria case data.

**Methods:**

This descriptive study documented the process of initiating and maintaining the PPM network in Lao PDR. Epidemiological data reported through the routine surveillance system from January 2009 to June 2016 were analyzed to illustrate the contribution of case reporting from the private sector.

**Results:**

A total of 2,301,676 malaria tests were performed in the PPM districts, which included all the PPM pharmacies and clinics (176,224, 7.7%), proportion of patients tested from 14,102 (4.6%) in 2009 to 29,554 (10.4%) in 2015. Over the same period of 90 months, a total of 246,091 positive cases (10.7%) were detected in PPM pharmacies and clinics (33,565; 13.6%), in the same districts as the PPM sites. The results suggest that the PPM sites contributed to a significant increasing proportion of patients positive for malaria from 1687 (7.4%) in 2009 to 5697 (15.8%) in 2015.

**Conclusions:**

Ensuring adequate and timely supplies of RDTs and ACT to PPM sites is critical. Frequent refresher training is necessary to maintain data quality, motivation and feedback. In the context of malaria elimination, the PPM initiative should be expanded further to ensure that all febrile cases seen through the private sector in malaria transmission areas are tested for malaria and treated appropriately. Results from the PPM must be integrated into a centralized registry of malaria cases that should prompt required case and foci investigations and responses to be conducted as part of elimination efforts.

## Background

In the Greater Mekong Sub-region (GMS), the private sector makes up a significant proportion of health services available to communities and often to hard-to-reach populations, making them important partners in the provision of appropriate diagnosis and treatment of malaria. Although the majority of malaria cases in Lao PDR is reported through public health facilities, the availability of anti-malarial drugs sold through pharmacies suggests that self-treatment of suspected malaria is likely more prevalent than previously acknowledged. This is one of the first examples in the GMS where a public–private partnership (PPM) for the provision of malaria diagnostic and treatment services through local registered pharmacies and clinics was comprehensively developed, implemented with all relevant public and private sector stakeholders and sustained with a high degree of reporting completeness over the last 8 years.

Public–private mix strategies have been used previously in public health interventions for tuberculosis [1, 2], neglected tropical diseases [3], as well as prevention of blindness [4], with some degree of success. There are limited instances demonstrating strong collaboration with the private sector for malaria diagnosis and treatment in published literature other than collaboration with the pharmaceutical industry for the provision of anti-malarial drugs [5]. Although a survey of national malaria elimination programmes in the Asia Pacific in 2015 [6] identified 5 out of 17 countries where anti-malarial treatments are available at public and/or private health facilities, published literature on initiation, processes, implementation, and evaluation of these public private initiatives are scarce.

The five southern provinces of Savannakhet, Saravane, Sekong, Attapeu, and Champasack account for more than 90% of reported malaria cases in Lao PDR. Of the total 147 districts in Lao PDR, more than half (53%) of all cases were reported from just 7 districts in Saravane and Champasack Provinces. The majority of cases are among males older than 5 years. The proportion of males with a positive malaria diagnosis increased from 46% in 2009 to 86% in 2014, suggesting a relationship between susceptibility of malaria infection and those most likely involved in the workforce, particularly forest workers and migrant labourers [7, 8].

Since 2004, artemisinin-based combination therapy (ACT), artemether–lumefantrine (CoArtem^®^), is the first-line treatment for *Plasmodium falciparum* malaria, the most prevalent species in the country [9]. Early diagnosis with rapid diagnostic tests (RDTs) and treatment with ACT was introduced in the public sector in 2005 and scaled up throughout the country. As per national policy, malaria microscopy is performed in all public hospitals, while RDTs are predominately used by village health workers (VHWs) and in health centres. Both diagnosis and treatment of malaria are provided free of charge in all public health facilities and by VHWs. By 2008, the use of RDTs and ACT had been rolled-out to 50 of 51 provincial facilities, 111 of 114 district facilities, 706 of 758 health centres, and 5241 villages with VHWs, representing approximately 85% of total villages. The PPM initiative sought to expand coverage of malaria services to the private sector while strengthening and maintaining quality-assured services in the public facilities and community levels.

## Private sector health providers

As of 2015, there are over 2132 private pharmacies and 222 private clinics registered throughout Lao PDR, most of which are found in urban areas and generally owned and serviced by public sector health practitioners after office hours and during weekends. Private pharmacies, categorized into three groups, are registered and regulated by the Food and Drug Department (FDD). Level 1 pharmacies are relatively larger shops, employ qualified pharmacists, and often act as pharmaceutical wholesalers; Level 2 pharmacies sell drugs wholesale but usually do not have full-time pharmacists on staff; and, Level 3 pharmacies are smaller, where the shop owner rents a licence from a registered pharmacist. Prior to the PPM scheme, registered Level 1, 2 and 3 pharmacies were not authorized to test for malaria, but could sell anti-malarials. By law, to date, unregistered outlets, such as village shops, grocery stores and informal drug stores and vendors, are not authorized to provide malaria case management services. The PPM initiative enabled the expansion of malaria service delivery points through selected registered pharmacies in the country.

## Methods

### PPM inception and process

The PPM pilot project was initiated in 2008 to engage the private sector by expanding access to early diagnosis and treatment of malaria was based on the World Health Organization’s public–private mix (PPM) strategy [10]. The main objectives were 1) to increase access of the population to ACT and RDTs, by making them available in the private sector; 2) to improve the quality of service provided in the private sector, particularly in following the national treatment guidelines; and, 3) to improve the reporting of malaria cases from the private sector.

**Fig. 1:**
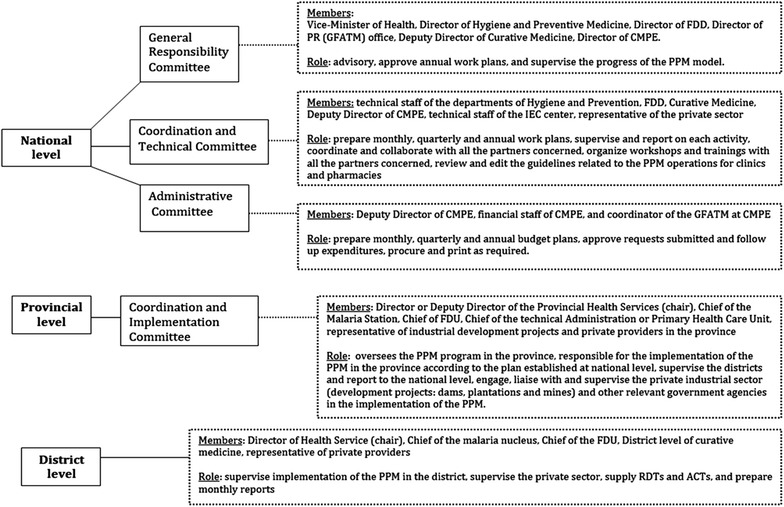
Coordinating and governance structure for the public–private mix strategy

The PPM initiative was developed through a series of consultations and consensus workshops involving representation from both government and private health sector (e.g., Lao Pharmacy Association, Physicians’ Association, pharmaceutical companies, and selected private providers). National, provincial and district PPM coordinating committees were established with terms of reference outlining their roles and responsibilities including the FDD and the Curative Health Department (CHD) of the Ministry of Health at central level (Fig. 1). The committees were also involved in determining the selection and inclusion criteria for PPM districts and private providers. Standard operating procedures (SOPs), including tools, reporting forms, operational and monitoring and evaluation plans were developed, and meetings at provincial levels were held to gain consensus.

As part of the preparatory phase of the project, the national malaria programme conducted a baseline mapping and assessment of eligible private providers in the 14 target districts of Luangnamtha, Savannakhet, Champassack and Attapeu Provinces. Registered private pharmacies (n = 151) and private clinics (n = 10) were identified: 82% (Level 3), 13% (Level 2), and 5% (Level 1). Among these, a total of 95 private providers (10 clinics and 85 pharmacies) were interviewed as part of the baseline survey. The majority (64%) of the pharmacies were run by pharmacists, 12% by mid-level pharmacist and 22% by either assistant doctors or nurses. Despite the relatively small sample size, knowledge of malaria diagnostic tools and appropriate treatment was particularly low among the private providers. Only one-third of the private clinics had either microscopes or RDTs for malaria diagnosis, and the drugs of choice cited by clinicians were chloroquine (43%), quinine (29%) and artesunate tablets (14%). The PPM programme was launched in 2008 in these 14 districts of 4 target provinces with participation of 151 registered private pharmacies and 10 private clinics.

### Incentives for pharmacies

Representatives from each participating private pharmacy or clinic received training on the diagnosis and treatment of malaria, and regular supervision from the district coordination and implementing teams. Anti-malarials and diagnostics were provided free-of-charge among PPM-registered private clinics and pharmacies through the existing logistics and supply channels of the public sector. Private providers were allowed to charge a maximum service fee of 2000 Lao Kip (LAK) (equivalent to 0.25 USD) for diagnosis with an RDT and 1000 LAK (equivalent to 0.12 USD) for treatment with Coartem (all ages/weights) if the test were positive. In addition, participating providers were supplied with signposts showing their participation in the PPM, and pharmacy staff received laboratory coats with PPM logos.

### Training, supervision and monitoring

Several training sessions were conducted in the months leading up to the launch of PPM in 2008 to prepare provincial and district coordinators and PPM site participants for their responsibilities. Provincial and district coordinators were trained in the management of PPM through SOPs, and PPM providers were trained in the diagnosis and detection of malaria using RDTs; the treatment of uncomplicated *P. falciparum* malaria with artemether–lumefantrine (Coartem™), including appropriate dosing, information to give patients at the time of treatment, drug and stock management, record-keeping and reporting requirements. Training curriculum for PPM providers was modified from existing national treatment guidelines. Reporting and stock management was based on SOPs for PPMs. Refresher trainings on how to perform RDTs correctly, administration of appropriate treatment dosages, dispensing practices and record keeping were conducted.

**Fig. 2:**
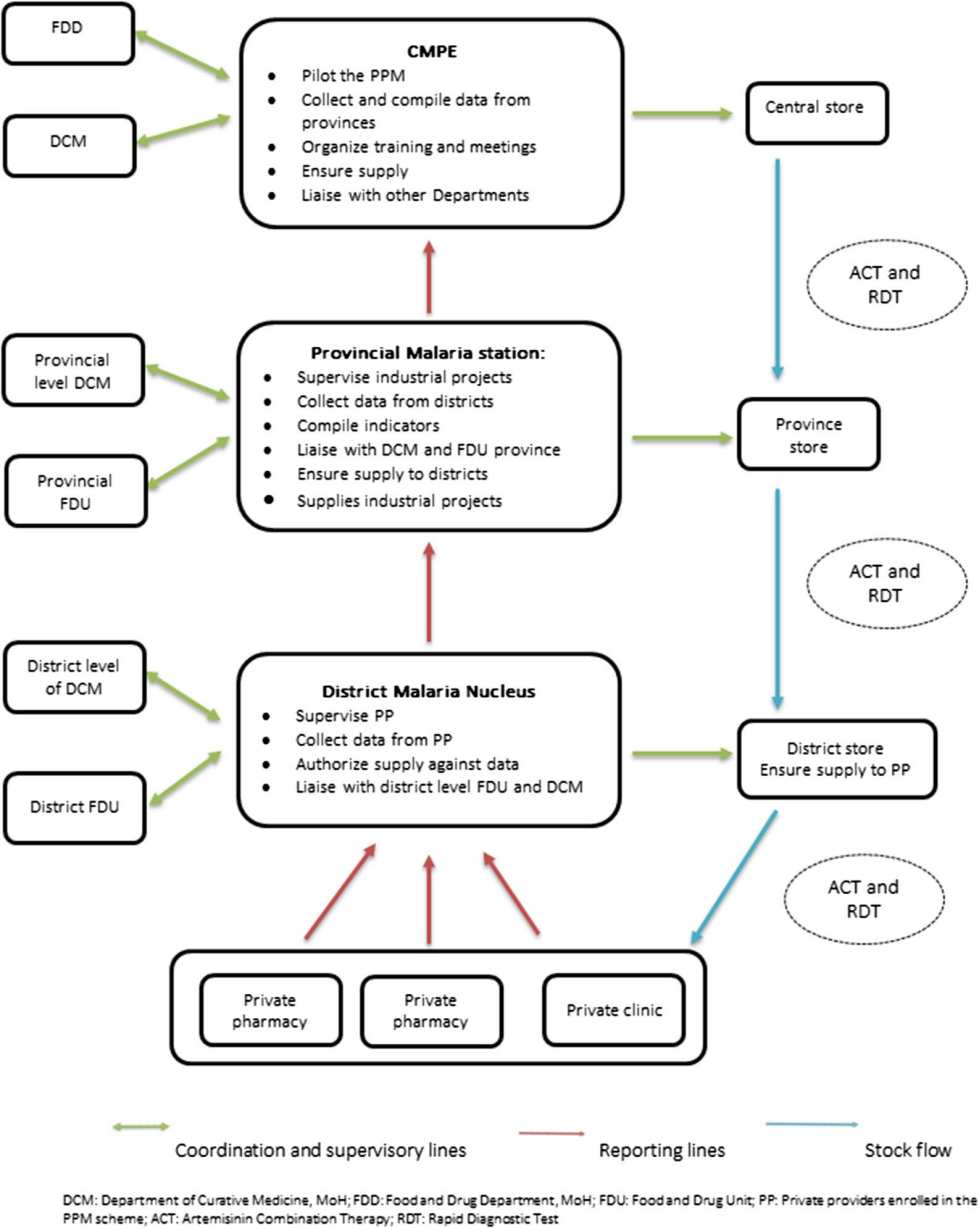
Reporting and coordination for public–private mix at national, provincial and district levels

Funds for regular supervision were allocated to provincial (quarterly supervision with malaria and FDD team from central level) and district (monthly supervision with malaria and FDU staff) staff to PPM sites (Fig. 2). A standardized form was used and feedback given to district staff and PPM sites at time of supervision visit, and a folder was kept at each PPM site recording all visits from the supervisor. Supervisions were done on average, once every quarter and focused on PPM sites where problems were detected either with reporting or performance of the provider. FDD/FDU monitoring of pharmacies was also done concurrently for 10 indicators of good pharmacy practices (GPP). Quality assurance/control guidelines on RDTs from the national programme involving random sampling were applied for both public and PPM sites. RDTs were sent to a WHO Collaborating Centre for quality testing. No issues on quality of RDTs supplied to the PPM sites were reported.

### Assessment and scale-up

**Fig. 3:**
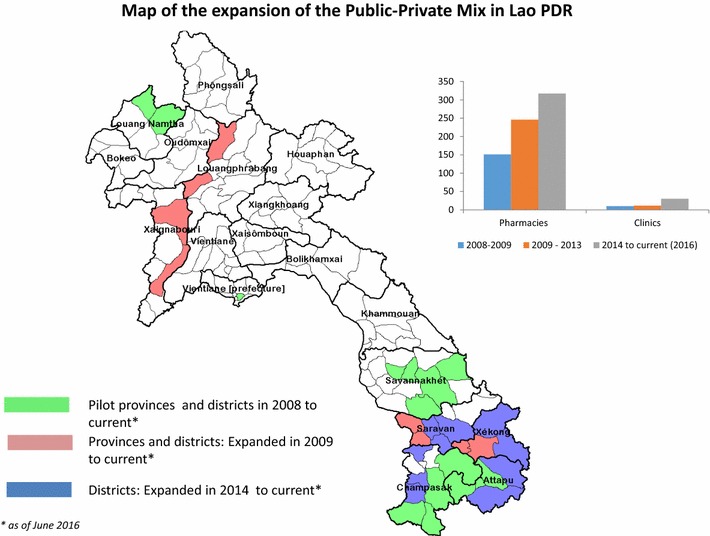
Map of the expansion of the public–private mix in Lao PDR

An external evaluation of the initial 4 PPM pilot provinces was conducted in October 2009 [11] to assess the PPM outcomes (i.e., case management, providers and beneficiaries) 1 year after the pilot introduction and to provide recommendations for future implementation. The assessment included field visits to provinces, districts and PPM sites, interviews, observations (observing PPM providers in clinics and pharmacies on ability to test and read results of the RDT correctly as patients present themselves at point in time), data collection and cross-checks of record keeping (cross-checking RDTs that tested positive and if they tallied with the number of malaria positive cases in the PPM record forms). While noting bottlenecks in stock management, the evaluation identified positive milestones towards achieving the three objectives of the PPM initiative and recommended that the PPM pilot should be expanded to other provinces. Consequently, a similar mapping as was done previously for the baseline in 2008 was undertaken in an additional 8 districts, which included 105 private pharmacies and 2 private clinics (Fig. 3). The PPM SOPs for central, provincial and district coordinators were revised in 2010 following the recommendations of the PPM evaluation and reporting forms; stock and patient forms were simplified and patient referral form was standardized.

By August 2010, there were a total of 8 provinces (22 districts, 246 pharmacies, and 11 private clinics) participating in the PPM project. In 2014, with the persistence of outbreaks in the southern provinces of Lao PDR affecting largely mobile populations, the national programme decided to scale-up the PPM initiative to an additional 10 districts in the south, enrolling an additional 71 private pharmacies and 19 private clinics. By June 2016, the PPM initiative was operational in a total of 32 districts of 8 provinces and included a network of 317 private pharmacies and 30 private clinics (Table 1, Fig. 3).

**Table 1:** Cumulative expansion of pharmacies and clinics in the public–private mix strategy from 2008 through June 2016

Province	District	2008		2009–2013		2014–2016	
		Number of pharmacies	Number of clinics	Number of pharmacies	Number of clinics	Number of pharmacies	Number of clinics
Louang Namtha							
	Namtha	17	3	17	3	13	3
	Sing	6	–	5	–	6	–
Louang Prabang							
	Nambak	–	–	18	–	17	–
	Chomphet	–	–	3	–	3	–
Xayabouly							
	Xayabouly	–	–	22	–	20	–
	Paklai	–	–	17	–	21	–
Savahnakhet							
	Sepon	7	–	7	–	8	3
	Palanxay	5	–	5	–	5	–
	Phin	7	3	7	3	7	4
	Atsapanthong	10	–	9	–	7	1
	Thapangthong	9	–	9	–	8	–
Saravanh							
	Kongsedone	–	–	13	2	12	2
	Lakonpheng	–	–	13	–	13	–
	Saravan	–	–	–	–	20	13
	Lao Ngam	–	–	–	–	10	–
	Vapi	–	–	–	–	9	–
Champassack							
	Khong	23	–	20	–	20	–
	Moonlapamok	12	–	12	–	12	–
	Phathompone	15	–	11	–	11	–
	Paksong	21	1	20	–	20	–
	Champasack	–	–	–	–	10	–
	Sukuma	–	–	–	–	10	–
	Sanasomboun	–	–	–	–	10	–
Sekong							
	Lamam	–	–	12	–	12	–
	Thateng	–	–	7	–	7	–
	Kalaem	–	–	–	–	2	–
	Dukchieng	–	–	–	–	2	–
Attapeu							
	Samakixay	12	3	12	3	9	4
	Xaysettha	4	–	4	–	6	–
	Sanxay	–	–	–	–	2	–
	Sanamxay	3	–	3	–	2	–
	Phouvong	–	–	–	–	3	–
Total		151	10	246	11	317	30

The study was conducted by the National Malaria Control Programme (NMCP), Ministry of Health, Lao PDR. This study did not involve the recruitment or testing and treatment of patients. The study reviewed and analysed epidemiological data and information available with the NMCP from January 2009 to June 2016. These included routine malaria surveillance data through the national Malaria Information System (MIS) accessed from the NMCP, Centre for Malariology, Parasitology and Entomology (CMPE), report from an external evaluation of the PPM initiative conducted in October 2009, routine supervision and monitoring reports to PPM sites over 2009–2015, as well as selected interviews with health care providers in both public health facilities and PPM registered pharmacies and clinics in 3 provinces conducted in 2013. In compliance with the national ethics committee, no ethical approval was required in this case. Data from an ACTwatch outlet survey conducted from November to December 2015 were also referenced. Details about the ACTwatch project and methodology have been published elsewhere [12]. The ACTwatch 2015 survey was conducted in 41 districts [13] in the southern five provinces (Attapeu, Champasak, Salavanh, Savannaket, Sekong) of which 25 districts were part of the PPM programme. This allowed for comparison of key indicators among pharmacies and private clinics in PPM and non-PPM districts.

## Results

### Malaria screening and treatment

**Fig. 4:**
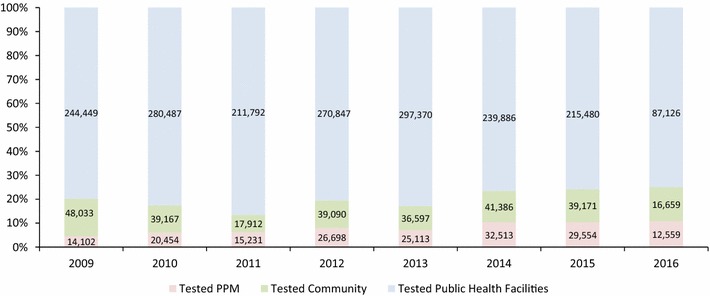
Proportion of malaria tests performed among public health facilities, community and public–private mix sites

**Fig. 5:**
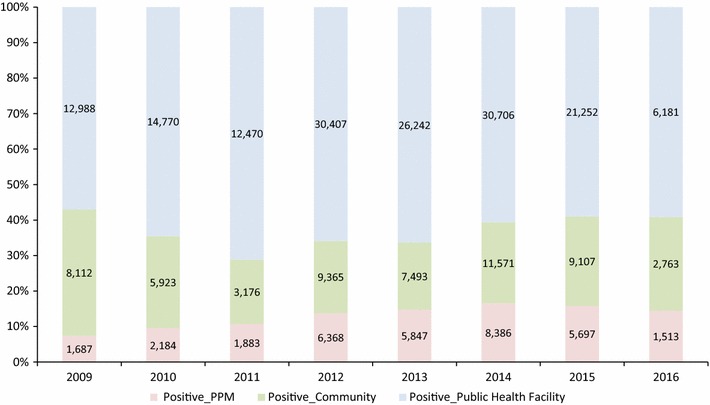
Proportion of malaria cases among public facilities, communities and public–private mix sites

**Fig. 6:**
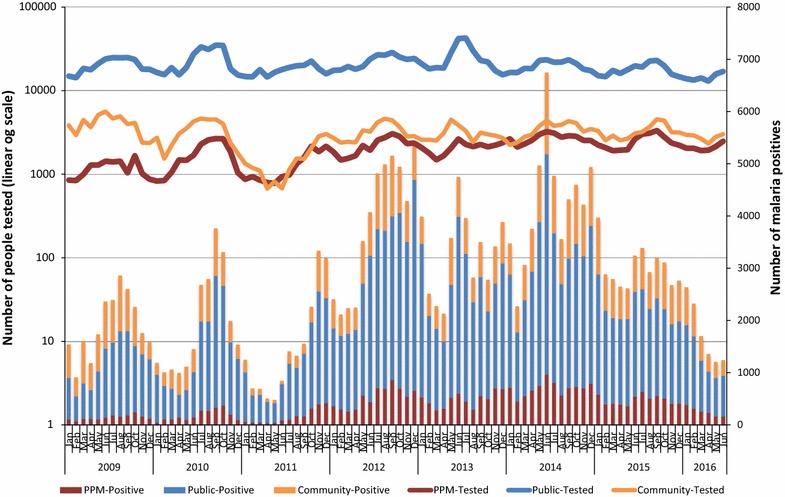
Trend of number of people tested and malaria positives among public health facilities, community and public–private mix sites

From January 2009 through June 2016 (90 months), a total of 2,301,676 tests were performed at PPM pharmacies and clinics (176,224, 7.7%), public health sector facilities (1,847,437, 80.3%) and community volunteers (278,015, 12.1%) in the same districts as the PPM sites (Fig. 4). Among the tests performed, a total of 246,091 (10.7%) malaria-positive cases were detected at PPM pharmacies and clinics (33,565, 13.6%), public health sector facilities (155,016, 63%) and community volunteers (57,510, 23.4%) in the same districts as the PPM sites (Fig. 5). The results suggest that the PPM sites contributed to a significant increasing proportion of patients tested from 14,102 (4.6%) in 2009 to 26,698 (8.0%) in 2012 and 29,554 (10%) in 2015 (R^2^ = 0.90) compared to the proportions for testing among public health facilities (2009: 244,449, 79.7% and 2015: 215,480, 75.8%) (R^2^ = 0.48) and in the community (2009: 48,033, 15.7% and 2015: 39,171, 13.8%) (R^2^ = 0.04) despite an overall decreasing trend of malaria burden and relatively stable screening rates among public health facilities, communities and PPM sites (Fig. 6). However, according to the ACTwatch survey in 2015 involving 236 public and 394 private sector outlets from 41 districts in the 5 southern provinces of Attapeu, Champasak, Salavanh, Savannaket, and Sekong (25 districts were part of the PPM programme), it was noted that testing availability was higher among pharmacies in PPM districts (71.6%) *versus* non-PPM districts (4.7%).

**Fig. 7:**
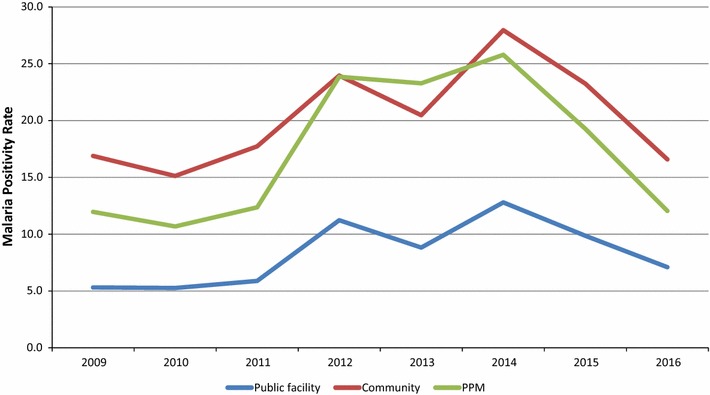
Trend of malaria positivity rates among public health facilities, community and public–private mix sites

Over the same period, the results also suggest that the PPM sites contributed to a significant increase in proportion of patients positive for malaria from 1687 (7.4%) in 2009 to 5697 (15.8%) in 2015, while numbers and proportion of malaria-positive cases in public health facilities increased marginally (2009: 12,988, 57% and 2015: 21,252, 58.9%) but decreased in the community (2009: 8112, 35.6% and 2015: 9107, 25.3%). The R-squared coefficients for proportional malaria positives: Facility (0.073), Community (0.080), and PPM (0.784) suggest a moderately strong correlation for an upward trend of proportional malaria positivity over time. Interestingly, the average annual malaria positivity rates at PPM sites (17.4%) were comparable to the malaria positivity rates found among community health workers (20.2%), both of which were observed to be significantly higher than the malaria positivity rate at public health facilities (8.3%) (Fig. 7).

Some PPM sites, particularly in the north of the country, rarely encountered stock-outs since there were fewer patients presenting with malaria symptoms and, therefore, utilized fewer RDTs and ACT, whereas PPM sites in the central and south required the use of more RDTs and ACT due to its higher malaria endemicity. Analysis of the ratio of RDT to ACT used by each district suggests that there was not preferential over-testing of RDTs among private providers.

### Referrals

PPM sites can refer more complicated malaria cases or other febrile illnesses to nearby health facilities or hospitals. Between January 2009 to December 2015, a total of 333 referrals were made from the PPM pharmacies and clinics in the 22 PPM districts. The majority of these referrals were made in Champasack Province (76%) and in Saravane Province (11%) in the southern part of the country. PPM sites know the signs of severe malaria and to refer patients with those signs to the nearest hospital. However, verification of registration of these referred patients in hospitals was not done in this study.

### Stock management

The PPM sites were supplied with RDTs and ACT through the same national malaria programme logistics and supply system for malaria commodities that is used for the public sector. As a result, the PPM does not have direct control over procurement, storage and distribution and was affected by many of the supply problems experienced throughout the country, largely, forecasting, procurement and distribution delays, which often originate and trickle-down from central level. These resulted in ACT reaching the PPM sites with a relatively short shelf life or the quantities planned were not necessarily available when needed. Shortages of ACT at central level and misallocation of quantities to the provinces also caused stock-outs at some PPM sites. It was often observed that stocks of RDTs and ACT were found at provincial and district levels, but these were not reaching the PPM pharmacy and clinic sites in a timely manner. Some PPM districts with relatively few patients could more effectively manage their RDTs and ACT from a single annual or bi-annual distribution.

## Discussion

Malaria case reporting from private providers in the GMS is notably limited, if reported at all. The PPM pilot was an attempt to expand access to quality-assured malaria diagnosis and treatment in Lao PDR through registered pharmacies and clinics. Access to anti-malarial treatment in the private sector already existed prior to the PPM initiative. Prior to the PPM, private sector treatment for malaria was at cost and not regulated (cost, type of anti-malarial, counterfeit and substandard). There was also no malaria diagnosis in private pharmacies and clinics. The PPM engaged private pharmacies and clinics based on criteria that related both to their geographical distribution, access patterns (baseline mapping was done) and malaria burden. The PPM initiative provided RDTs to improve quality of diagnosis and ACT to improve quality of drugs and treatment while at the same allowing pharmacies to profit minimally from this engagement. Through the branding of the PPM initiative, pharmacies and clinics are able to provide a standard of care that otherwise would not have been monitored.

The increasing trend of malaria tests performed as well as malaria test positivity rates detected among PPM sites over the past 7 years compared to public health facilities and even community health workers is encouraging. However, the intention is not to divert treatment-seeking and service provision from public health facilities and community health workers to the private providers. Similar support to public sector facilities to ensure adequate supplies of commodities are available, provision of quality services, and regular monitoring and supervision should also be strengthened.

The use of PPM for malaria in a moderately low malaria transmission setting, such as in the northern provinces of Lao PDR, presents an interesting scenario to consider the cost-effectiveness of the PPM strategy. With fewer and fewer suspected malaria patients presenting at public and private sector facilities, the likelihood that the patient is suffering from another ailment is more likely than not. It will be useful to assess the cost-effectiveness of implementation of the PPM strategy in different epidemiological settings to determine the value-added for such interventions especially in the context of elimination of malaria. Private providers will need to be equipped with the appropriate diagnostic tools and treatments for these other illnesses, and be able to refer severe cases to local hospitals. From the current referral system, it is unclear what proportion of these referral patients actually reaches the hospital.

Furthermore, it is important to strike the right balance for private providers to continue to engage in the PPM. By subsidizing the costs of RDTs and ACT for the private sector, as well as allowing private providers to charge a nominal fee for these services, the PPM strategy attempts to provide adequate incentives for participating providers. It appears that PPM sites are not solely motivated by the additional sales of RDTs and ACT, but rather could be benefiting more substantially from the sale of other medicines to treat the non-malaria illness as well as supplementary medicines, such as anti-pyretics and vitamins. Addressing issues of satisfaction, motivation, feedback, and adherence are important to ensure longer-term commitment and engagement of these private providers, all of which will require further in-depth qualitative assessments.

There were some limitations to this project that are worth noting. Firstly, non-registered pharmacies were not included in the network. The proportion of people who obtain their anti-malaria drugs from non-registered sources in Lao PDR is still unknown, but it is hoped that the PPM would reduce the demand for and push out itinerant vendors. Secondly, the increasing number of cases seen in the private sector is a positive sign, but should not be seen at the expense of the public sector. In fact, regulatory changes were needed at the inception to allow even registered private providers to test and treat for malaria. Both public and private sectors need to be strengthened with fully trained health providers equipped with readily available and easy-to-use diagnostics (i.e., RDTs) and quality-assured ACT. Despite the seemingly positive trend of ‘additional’ malaria cases detected through PPM, the issue of double-counting remains an important one that warrants further investigation. This could result in counting the same individual once through the PPM reporting system and again through the public sector when the patient is referred to the hospital. Further investigation, including more in-depth qualitative assessments of the patient and follow-up, should be considered in further evaluations. Thirdly, the current lack of individual-level data that are ultimately aggregated up to the national level is a missed opportunity for more detailed information regarding patients’ origin and travel history which would be useful to inform better strategies for improving access among mobile and migrant populations.

Despite these limitations, there were a few lessons learned that could have further improved the performance and management of PPM. Firstly, the national programme should consider centralizing the data collected from individuals presenting at PPM sites by maintaining the line lists. Moving towards malaria elimination, malaria programmes should be requiring that all malaria cases are line-listed with contact information, including address, for follow-up. Currently, this information is collected by private pharmacies, but is lost when aggregated at the district level. Secondly, PPM data collected should be integrated with the routine malaria information system, including logistics and stock management as part of the national malaria surveillance system. This will vastly improve epidemiological and stock monitoring at the periphery, as well as potentially enable better tracking and follow-up of referral patients. Thirdly, it will be critical to maintain the motivation of the PPM sites through consistent feedback, regular refresher training, as well as standardization of the PPM branding and behaviour change communications.

## Conclusions

Despite some issues with stock-outs of RDTs and ACT in some provinces and districts, the PPM programme in Lao PDR appeared to be quite successful and could be a model for other countries in the GMS. The planning process was inclusive, participatory and involved all relevant sectors of the public health and regulatory system, private health sector stakeholders and providers. PPM site selection demonstrated responsiveness to programme needs, malaria epidemiology and took into account the realities in the field. Responsibility for programme implementation was largely focused at district level and helped to build capacity in programme management.

The private sector remains nascent in Lao PDR but stands to benefit from the overall economic growth in the region. Following the models utilized for other public health interventions such as for TB’s PPM-DOTS, it might be worth considering expanding the use of an integrated-PPM for other illnesses in the future, particularly for sites that may not see malaria as one of the top priorities. In addition, in areas with low malaria transmission, it may be more important to consider these PPM sites potentially as sentinel surveillance and monitoring sites for the detection of malaria outbreaks, provided data are available and analysed quickly for action.

This is one of the first examples of a public–private partnership for the diagnosis and treatment of malaria that was completely led and owned by the Ministry of Health. Certainly there are areas that could be improved, but this has been a relatively large scale and comprehensive effort to reach out to the elusive private sector through an official public–private mix strategy. With improved and more inclusive data from the private sector, Lao PDR is one step closer toward their malaria elimination goals.

## Abbreviations

ACT: artemisinin-based combination therapy
AL: artemether–lumefantrine
CHD: Curative Health Department
CIEH: Centre for Information, and Education for Health
CMPE: Centre for Malariology, Parasitology and Entomology
DHC: Department for Health Care
DHIS2: District Health Information System
DOTS: directly observed treatment, short course
FDD: Food and Drug Department
FDU: Food and Drug Unit
GFATM: Global Fund to Fight AIDS, Tuberculosis, and Malaria
GMS: Greater Mekong Sub-region
LLIN: long-lasting insecticidal net
M&E: monitoring and evaluation
oAMT: oral artemisinin monotherapy
PPM: public private mix
PR: Principal Recipient of the Global Fund to Fight AIDS, Tuberculosis, and Malaria
RDT: rapid diagnostic test
SOP: standard operating procedures
TB: tuberculosis
VHW: village health worker
